# Ectopic Bone Tissue Engineering in Mice Using Human Gingiva or Bone Marrow-Derived Stromal/Progenitor Cells in Scaffold-Hydrogel Constructs

**DOI:** 10.3389/fbioe.2021.783468

**Published:** 2021-11-30

**Authors:** Siddharth Shanbhag, Carina Kampleitner, Samih Mohamed-Ahmed, Mohammed Ahmad Yassin, Harsh Dongre, Daniela Elena Costea, Stefan Tangl, Mohamad Nageeb Hassan, Andreas Stavropoulos, Anne Isine Bolstad, Salwa Suliman, Kamal Mustafa

**Affiliations:** ^1^ Center for Translational Oral Research (TOR), Department of Clinical Dentistry, Faculty of Medicine, University of Bergen, Bergen, Norway; ^2^ Department of Immunology and Transfusion Medicine, Haukeland University Hospital, Bergen, Norway; ^3^ Ludwig Boltzmann Institute for Traumatology, The Research Center in Cooperation With AUVA, Vienna, Austria; ^4^ Karl Donath Laboratory for Hard Tissue and Biomaterial Research, University Clinic of Dentistry, Medical University of Vienna, Vienna, Austria; ^5^ Austrian Cluster for Tissue Regeneration, Vienna, Austria; ^6^ Gade Laboratory for Pathology, Department of Clinical Medicine, Faculty of Medicine, University of Bergen, Bergen, Norway; ^7^ Centre for Cancer Biomarkers (CCBIO), Faculty of Medicine, University of Bergen, Bergen, Norway; ^8^ Department of Periodontology, Faculty of Odontology, Malmö University, Malmö, Sweden; ^9^ Division of Conservative Dentistry and Periodontology, University Clinic of Dentistry, Medical University of Vienna, Vienna, Austria

**Keywords:** xeno-free, platelet lysate, MSc, spheroid culture, bone tissue engineering

## Abstract

Three-dimensional (3D) spheroid culture can promote the osteogenic differentiation and bone regeneration capacity of mesenchymal stromal cells (MSC). Gingiva-derived progenitor cells (GPC) represent a less invasive alternative to bone marrow MSC (BMSC) for clinical applications. The aim of this study was to test the *in vivo* bone forming potential of human GPC and BMSC cultured as 3D spheroids or dissociated cells (2D). 2D and 3D cells encapsulated in constructs of human platelet lysate hydrogels (HPLG) and 3D-printed poly (L-lactide-co-trimethylene carbonate) scaffolds (HPLG-PLATMC) were implanted subcutaneously in nude mice; cell-free HPLG-PLATMC constructs served as a control. Mineralization was assessed using micro-computed tomography (µCT), histology, scanning electron microscopy (SEM) and *in situ* hybridization (ISH). After 4–8 weeks, µCT revealed greater mineralization in 3D-BMSC vs. 2D-BMSC and 3D-GPC (*p* < 0.05), and a similar trend in 2D-GPC vs. 2D-BMSC (*p* > 0.05). After 8 weeks, greater mineralization was observed in cell-free constructs vs. all 2D- and 3D-cell groups (*p* < 0.05). Histology and SEM revealed an irregular but similar mineralization pattern in all groups. ISH revealed similar numbers of 2D and 3D BMSC/GPC within and/or surrounding the mineralized areas. In summary, spheroid culture promoted ectopic mineralization in constructs of BMSC, while constructs of dissociated GPC and BMSC performed similarly. The combination of HPLG and PLATMC represents a promising scaffold for bone tissue engineering applications.

## Introduction

Adult mesenchymal stromal cells (MSC) are increasingly being used in bone tissue engineering (BTE) for the reconstruction of clinically challenging bone defects, and to overcome the limitations of existing bone-substitute materials (Shanbhag et al., 2019). Although MSC derived from bone marrow (BMSC) are the most widely tested, progenitor cells from other tissues requiring less-invasive harvesting, e.g., oral tissues, are being explored (Sharpe, 2016; Pittenger et al., 2019). Gingiva, in particular, can be harvested with minimal morbidity and contains a subpopulation of multipotent progenitor cells (GPCs), which demonstrate an MSC-like phenotype, immunomodulatory properties, and osteogenic potential both *in vitro* and *in vivo* (Fournier et al., 2010; Mitrano et al., 2010; Tomar et al., 2010).

A critical aspect in the clinical translation of cell therapies is the use of safe and standardized culture conditions resulting in safe-to-use cell constructs. Exclusion of animal-derived supplements, e.g., fetal bovine serum (FBS), in *ex vivo* culture systems is considered important to facilitate clinical translation of cell therapies and is also a recommendation by regulatory health authorities (Bieback et al., 2019). Pooled human platelet lysate (HPL) has been identified as the optimal “xeno-free” supplement for MSC culture, with particular benefits for BTE by promoting MSC osteogenic differentiation (Fekete et al., 2012; Shanbhag et al., 2017). We have recently reported that HPL cultured GPC and BMSC demonstrate superior proliferation, osteogenic gene expression and *in vitro* mineralization vs. corresponding FBS-based cultures (Shanbhag et al., 2020a; Shanbhag et al., 2020b).

Compared to two-dimensional (2D) monolayer cultures, the self-assembly or aggregation of MSC into 3D spheroids is mediated by unique cell-cell and cell-extracellular matrix (ECM) interactions, biomechanical cues and activated signaling pathways, simulating more closely the *in vivo* microenvironment (Sart et al., 2014; Cesarz and Tamama, 2016). Several studies have reported that, compared to conventional 2D monolayers, spheroid MSC show enhanced “stemness”, differentiation capacity, paracrine activity and immunomodulatory potential (Kale et al., 2000; Follin et al., 2016; Petrenko et al., 2017). We have recently reported that the expressions of several genes associated with self-renewal and osteogenic differentiation were significantly enhanced in xeno-free 3D spheroid vs. 2D monolayer cultures of GPC and BMSC, independent of osteogenic induction via media supplements (Shanbhag et al., 2020b). GPC and BMSC spheroids also demonstrated *in situ* mineralization and ECM formation following *in vitro* osteogenic induction, altogether, suggesting a promising potential for use in BTE (Shanbhag et al., 2020b).

Traditional cell delivery methods involve direct seeding of cells on the surface of biomaterial scaffolds before *in vivo* transplantation (Shanbhag and Shanbhag, 2015). However, this may not be optimal for MSC spheroids where the 3D structure is lost by direct seeding, thus potentially compromising their efficacy. To preserve the 3D structure, encapsulation of spheroids in hydrogel scaffolds maintains their 3D assembly and represents an effective delivery system (Murphy et al., 2014; Murphy et al., 2015; Ho et al., 2018). Since HPL is increasingly being used for clinical-grade MSC culture, extending its application as a hydrogel scaffold represents a clinically relevant and cost-effective strategy (Robinson et al., 2016). Additionally, using 3D-printing technology, pliable scaffolds of novel copolymers, e.g., poly (L-lactide-co-trimethylene carbonate) (PLATMC) (Jain et al., 2020), can be custom designed to support the cell-hydrogel constructs in non-contained critical-size bone and/or periodontal defects (Hassan et al., 2019; Yamada et al., 2021). As a preliminary step, the regenerative potential of tissue engineered constructs is frequently tested in ectopic, e.g., subcutaneous or intramuscular, sites (Scott et al., 2012). The absence of local osteogenic cells and stimuli surmises that any observed mineralization is from exogenous origins and/or stimuli. Therefore, the objective of the present study was to compare the potential of xeno-free GPC and BMSC, as dissociated cells (2D) or spheroids (3D), encapsulated in constructs of HPL hydrogels (HPLG) and PLATMC (HPLG-PLATMC), for ectopic BTE in a subcutaneous immunocompromised mouse model.

## Materials and Methods

### Cell Culture

The use of human cells and tissues was approved by the Regional Committees for Medical Research Ethics (REK) in Norway (2013-1248/REK-sør-øst C and 2016-1266/REK-nord) and obtained following appropriate informed consent. Bone marrow aspirates were obtained from three donors (one female and two males; 8–10 years) undergoing corrective surgery at the Department of Plastic Surgery, Haukeland University Hospital, Bergen, Norway. Gingival biopsies were collected from three systemically healthy, non-smoking patients (two females and one male; 18–31 years) undergoing dental surgery at the Department of Clinical Dentistry, University of Bergen, Bergen, Norway. BMSC and GPC were isolated as previously described (Mohamed-Ahmed et al., 2018; Shanbhag et al., 2020b). Briefly, primary monolayer cultures of GPC and BMSC were separately established in growth media comprising Dulbecco’s Modified Eagle’s medium (DMEM; Invitrogen, Carlsbad, CA, United States) supplemented with 5% (v/v) HPL (Bergenlys^®^, Bergen, Norway), 1% (v/v) penicillin/streptomycin (GE Healthcare, South Logan, UT, United States) and 1 IU/ml heparin (Leo Pharma AS, Lysaker, Norway). Cells were sub-cultured (4,000 cells/cm^2^) and expanded in humidified 5% CO_2_ at 37°C. Characterization of monolayer GPC and BMSC according to the “minimal MSC criteria” (Dominici et al., 2006), i.e., plastic adherence, stromal-like immunophenotype and multi-lineage differentiation potential, has been reported elsewhere (Shanbhag et al., 2020a; Shanbhag et al., 2020b).

To generate 3D spheroids, passage-2 dissociated monolayer GPC and BMSC (*n* = 3 donors, pooled) were separately seeded in microwell-patterned 24-well plates (Kugelmeiers Ltd., Erlenbach, CH); after 24 h, aggregate spheroids of ∼1000 cells were formed via guided self-assembly (Shanbhag et al., 2020b). Characterization of GPC and BMSC spheroids based on gene expression, cytokine secretion and *in vitro* mineralization, has been reported elsewhere (Shanbhag et al., 2020b).

### Fabrication of HPLG-PLATMC Constructs

PLATMC scaffolds were produced as described elsewhere (Jain et al., 2020). Briefly, a 3D CAD model was designed using the Magics^®^ software integrated with a 3D-Bioplotter^®^ (both from EnvisionTEC, Gladbeck, Germany). Granules of medical-grade PLATMC (RESOMER^®^ LT-706-S 70:30, Evonik GmBh, Essen, Germany) were loaded into the printer cartridge (pre-heated to 220°C) and rectangular sheets of three layers with an orientation of 0°–90°−0° were printed at 190°C with an inner nozzle diameter of 400 μm and strand spacing of 0.7 mm. Disc-shaped scaffolds measuring 6 mm × ∼1.2 mm were punched out and placed in 48-well plates. Prior to use in experiments, the scaffolds were sterilized by soaking in 70% ethanol for 30 min, followed by washing with phosphate-buffered saline (PBS, Invitrogen) and 2 h exposure to UV light.

**FIGURE 1:**
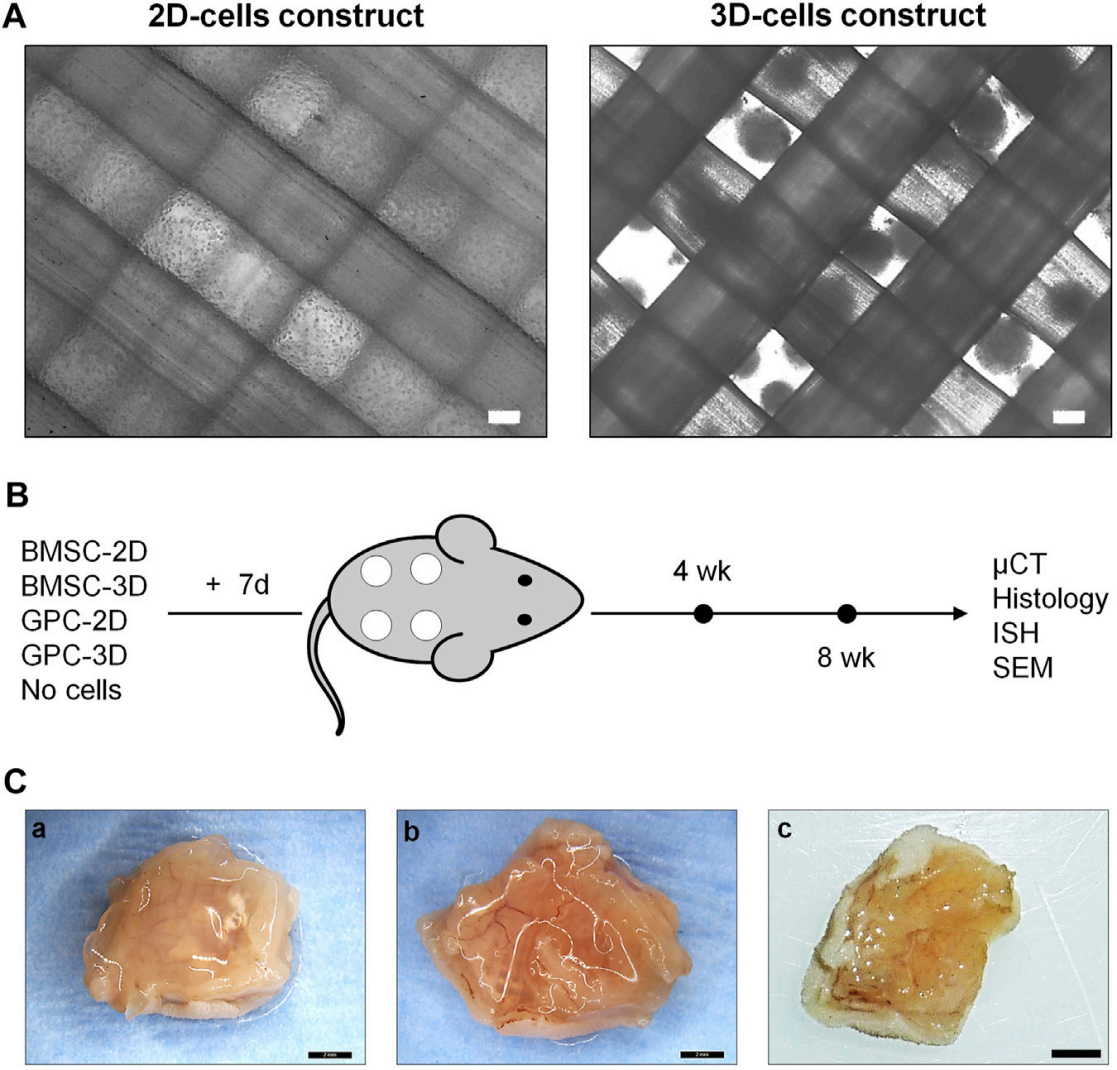
Study design. **(A)** Representative phase microscopic images of 2D (single cells) and 3D (spheroids) cell constructs; scale bars 100 μm. **(B)** Schema of study design, experimental groups and outcomes; constructs were cultured *in vitro* in osteogenic induction medium for 7 days prior to implantation (+7 days). **(C)** Representative macroscopic images of 8-weeks tissue specimens containing BMSC (a), GPC (b) or cell-free constructs (c); scale bars 2 mm.

To avoid direct seeding on scaffolds and aiming to preserve the morphology of 3D spheroids, HPLG was added to the construct. To prepare HPLG, sterile-filtered HPL (same as in growth media) was supplemented with 20 mg/ml fibrinogen (Sigma-Aldrich, St. Louis, MO, United States) to increase the stiffness and mechanical properties of the hydrogel (Murphy et al., 2015). Gelation was achieved by adding a “thrombin solution” containing 1 IU/ml human thrombin and 1 TIU/ml aprotinin in 20 mM CaCl_2_ solution (all from Sigma-Aldrich), followed by incubation at 37°C for 15 min. To prepare the (cell-free) constructs, the HPL and thrombin solutions were mixed and 50 μl were quickly seeded on pre-wetted scaffolds. To prepare cell-loaded constructs, equal numbers of passage-2 2D or 3D GPC or BMSC were uniformly suspended in fibrin supplemented HPL, mixed with thrombin solution and seeded on scaffolds (2 × 10^6^ cells in 50 μl) as described above. Cell distribution within the constructs was observed under a light microscope (Nikon Eclipse TS100, Tokyo, Japan) (Figure 1A). Constructs were cultured in osteogenic induction media, i.e., growth media supplemented with final concentrations of 0.05 mM L-ascorbic acid 2-phosphate, 10 nM dexamethasone and 10 mM β glycerophosphate (all from Sigma-Aldrich), for 1 week prior to *in vivo* implantation.

### Ectopic Implantation in Nude Mice

Animal experiments were approved by the Norwegian Animal Research Authority (Mattilsynet; FOTS-18738) and reported in accordance with the ARRIVE guidelines for all relevant items (Kilkenny et al., 2010; Berglundh and Stavropoulos, 2012). Twenty female athymic nude mice (Rj:Athym-Foxn1nu/nu, Janvier Labs, France), 7-weeks-old and weighing 19.4 ± 1.12 g, were used. Animals were housed in stable conditions (22 ± 2°C) with a 12 h dark/light cycle and *ad libitum* access to food and water. Animals were allowed to acclimatize for 1 week prior to experiments and were regularly monitored for signs of pain/infection, food intake and activity during the entire experimental period.

Pre-operatively, animals were anesthetized with a mixture of sevoflurane (Abbott Laboratories, Berkshire, United Kingdom) and O_2_ using a custom-made mask. Following anaesthesia, two 1-cm incisions were made in the midline of the dorsum, and four subcutaneous pouches were created using blunt dissection. Next, four constructs per animal containing either suspension [2 × 10^6^ 2D-BMSC or 2D-GPC], spheroid [2 × 10^6^ 3D-BMSC or 3D-GPC] or no cells were randomly implanted in the pouches (5 groups; *n* = 8 constructs per group per time point). GPC and BMSC were never implanted in the same animals. Post-operatively, the skin was sutured (Vicryl, Ethicon, Somerville, NJ, United States) and animals were injected subcutaneously with buprenorphine (Temgesic 0.03 mg/kg, Schering-Plough, United Kingdom) for up to 2 days thereafter. After 4 or 8 weeks, the animals were euthanized with an overdose of CO_2_ and constructs were harvested. The primary outcome, i.e., ectopic mineralized tissue formation, was assessed via micro-computed tomography (μCT) and histology. Secondary outcomes included identification of transplanted human cells by *in situ* hybridization (ISH) and assessment of mineralized ultrastructure by scanning electron microscopy and energy dispersive x-ray spectroscopy (SEM/EDX) analysis. Animals were coded via ear clips and identified by numbers for all subsequent handling/analyses to facilitate blinding of personnel.

### μCT

Immediately after euthanasia, the specimens were harvested along with the overlying skin and underlying muscle tissues and fixed in 10% buffered formalin (Sigma-Aldrich). Specimens were scanned using a SkyScan 1172 μCT scanner (Bruker, Kontich, Belgium) with an X-ray source of 60 kV/200 μA and 0.5 mm aluminum filter for a 10 µm resolution. Scans were reconstructed by applying a standardized volume of interest (5 mm × 1 mm to exclude the tissue margins) and a global grey threshold of 110–255 using the CTAn v.1.18 software (Bruker). Quantification of mineralization as a ratio of the total construct volume (MdV/TV) was performed in a blinded fashion using the CTAn software (Bruker).

### Histology

Specimens were processed for histology by both decalcified (paraffin-embedded) and undecalcified (resin-embedded) methods. Selected specimens were decalcified in 20% ethylenediaminetetraacetic acid solution (EDTA; Sigma-Aldrich) for 7 days. Next, formalin-fixed tissues were dehydrated in ascending grades of alcohol and embedded in paraffin (FFPE) or light-curing resin (RE; Technovit 7200 + 1% benzoyl peroxide, Kulzer & Co., Wehrheim, Germany). FFPE tissue sections were cut (∼5 µm) and stained with hematoxylin and eosin, Alizarin red S (Sigma-Aldrich) or Trichrome dyes (Roche Diagnostics, Oslo, Norway); Alizarin red staining was performed on undecalcified FFPE sections. RE specimens were further processed using EXAKT cutting and grinding equipment (EXAKT Apparatebau, Norderstedt, Germany) and thin ground sections (∼100 µm) were stained with Levi-Lazko dye (Morphisto GmbH, Frankfurt, Germany). FFPE and RE sections were scanned and digitized using a Nanozoomer XR (Hamamatsu, Photonics Ltd., Hertfordshire, United Kingdom; ×40 magnification) and Olympus BX61VS system (DotSlide 2.4; Olympus, Japan, Tokyo, ×20 magnification), respectively. Quantification of total collagen (area in µm^2^) in Trichrome stained FFPE sections was performed using QuPath open-source image analysis software (Bankhead et al., 2017).

### ISH

Detection of transplanted human cells was performed using ISH for the human specific repetitive *Alu* sequence, which comprises approximately 5% of the total human genome (Mankani et al., 2007). ISH was performed using the RNAscope 2.5 High-Definition Brown Assay according to the manufacturer’s instructions (all reagents and probes from Advanced Cell Diagnostics, Newark, CA, United States). Briefly, tissue slides were baked at 60°C for 1 h followed by deparaffinization in 100% xylene twice for 5 min each and two changes of 100% ethanol. The slides were treated with an endogenous peroxidase-blocking reagent, incubated for 15 min in boiling 1× target retrieval solution and treated with protease digestion buffer for 30 min at 40°C. The slides were then incubated with the target *Alu* probe for 2 h at 40°C, followed by signal amplification as detailed in the manufacturer’s guide. For colorimetric detection, 3,3′-Diaminobenzidine (DAB) was applied for 5 min at RT followed by counterstaining with hematoxylin. A peptidylprolyl isomerase B (*PPIB*) Positive Control Probe was used to validate the assay. Quantification of brown stained *Alu +* cells in ISH sections was performed using the QuPath software (Bankhead et al., 2017).

### SEM

Ultrastructure of mineralization in the undecalcified ground sections was analyzed using SEM and EDX. Briefly, the slides were sputter coated with carbon and imaged at a voltage of 15 kV with an electron microscope (Supra 55VP, Carl Zeiss, Oberkochen, Germany). EDX analysis was performed using the Pathfinder software (Thermo Scientific) and atomic weight percentages of various elements such as calcium (Ca) and phosphorous (P) were automatically calculated. EDX analysis was performed at least three different regions of the mineralized tissues in each section. Sections of histologically validated ectopic bone from a previous study in mouse intramuscular sites were analyzed as positive controls.

### Statistical Analysis

Statistical analysis was performed using the Prism 9.0 software (GraphPad Software, San Diego, CA, United States). Data are presented as means ± SD, unless specified. Normality testing was performed using the Shapiro-Wilk test. The student *t* test, Mann-Whitney U test and one-way analysis of variance (ANOVA), followed by a post-hoc Tukey’s (parametric) or Dunn’s test (non-parametric) for multiple comparisons, were applied as appropriate, and *p* < 0.05 was considered statistically significant.

## Results

### General Outcomes

HPLG-PLATMC constructs containing equal numbers of 2D or 3D GPC or BMSC were implanted subcutaneously in nude mice (Figures 1A,B). One animal died 2 days postoperatively due to an eye infection unrelated to the implants and was excluded from the analysis. All other animals recovered from surgery and no adverse events were recorded. Constructs were analyzed after 4 weeks [2D-BMSC (*n* = 8), 3D-BMSC (*n* = 8), 2D-GPC (*n* = 6), 3D-GPC (*n* = 6), cell-free (*n* = 8)] or 8 weeks [2D-BMSC (*n* = 8), 3D-BMSC (*n* = 8), 2D-GPC (*n* = 8), 3D-GPC (*n* = 8), cell-free (*n* = 8)]. No signs of inflammation were observed on either the skin or muscle surface. Abundant blood vessels were observed in the muscle layer directly underlying the constructs in all groups.

### Spheroid Culture of BMSC Promoted Ectopic Mineralization

**FIGURE 2:**
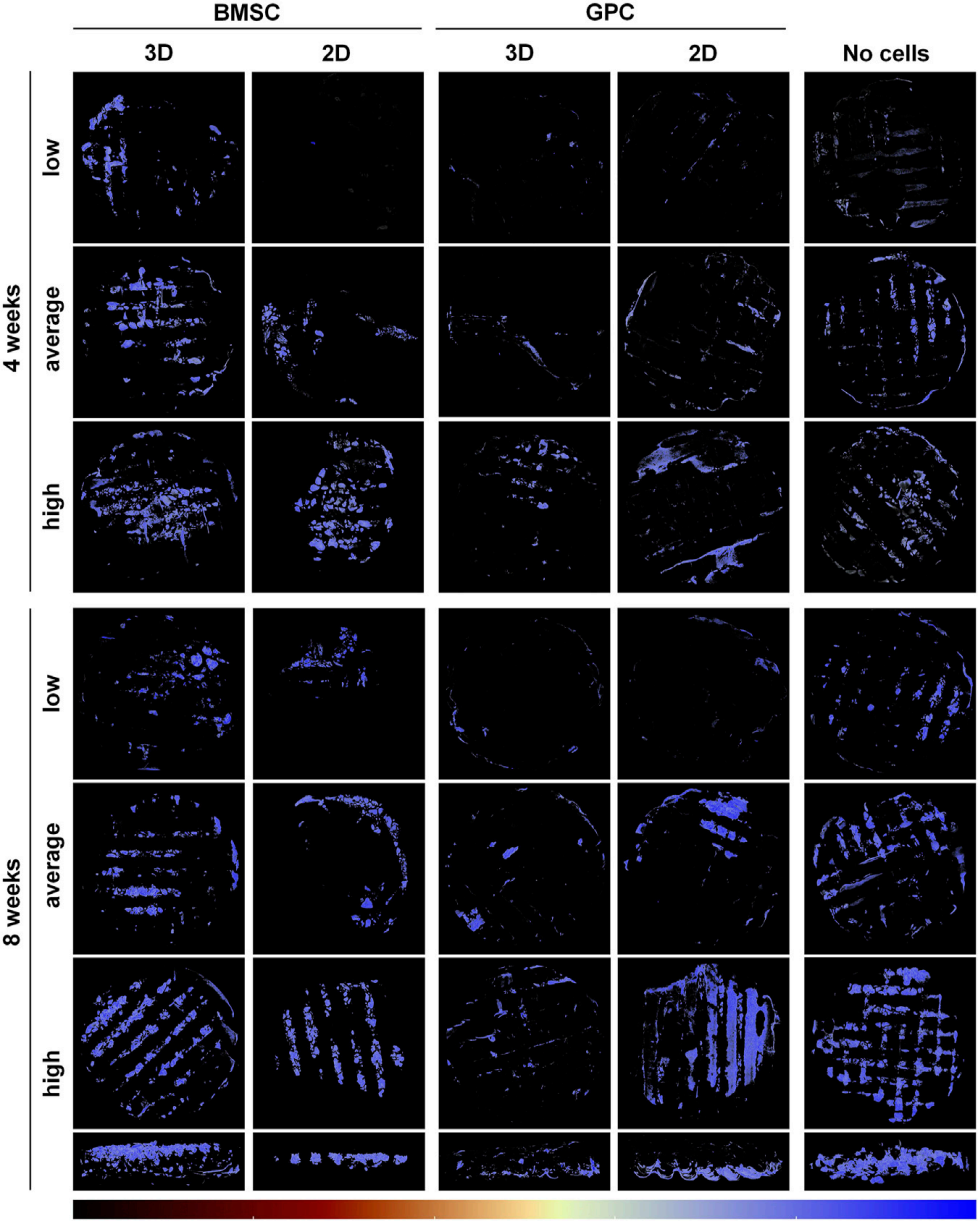
μCT analysis of ectopic mineralization. Representative reconstructed images showing “low, average and high” degrees of mineralization in BMSC, GPC and control constructs (no cells) after 4 and 8 weeks; the bottom row shows cross-sectional views of the corresponding “high” constructs at 8 weeks. The color bar indicates relative mineral density from minimum (black) to maximum (blue).

**FIGURE 3:**
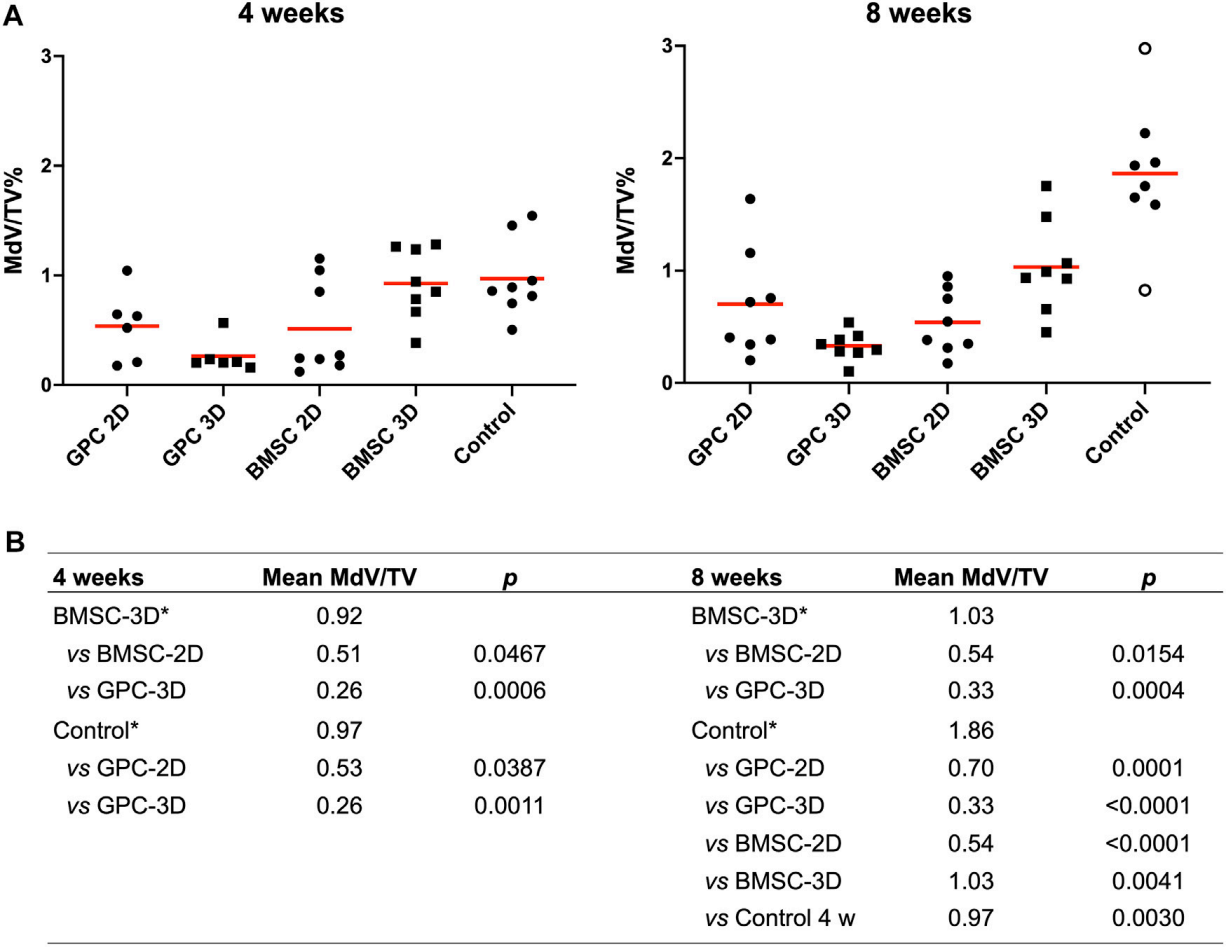
Quantification of mineralization by μCT. **(A)** Percentage mineralization in BMSC, GPC and control constructs (no cells) after 4 and 8 weeks; MdV/TV, mineral volume/total construct volume; data represent means; o represents outliers. **(B)** Inter-group comparisons showing statistically significant differences (*p* < 0.05); * reference group in the analysis (Kruskal-Wallis one-way ANOVA).

μCT analysis revealed mineralization of varying degrees in all groups. The pattern of mineralization typically followed the scaffold architecture, i.e., along the surface of the printed filaments in between the pores (Figure 2). Cross-sectional images demonstrated mineralization throughout the entire thickness of the construct. Significantly greater mineralization (MdV/TV) was observed in 3D-BMSC vs. 2D-BMSC constructs after 4 (0.92 ± 0.32 vs. 0.51 ± 0.42; *p* = 0.046) and 8 weeks (1.03 ± 0.41 vs. 0.54 ± 0.28; *p* = 0.015) (Figures 3A,B). In the case of GPC, a non-significant trend for greater MdV/TV was observed in 2D-GPC vs. 3D-GPC constructs at 4 (0.53 ± 0.32 vs. 0.26 ± 0.15; *p* > 0.05) and 8 weeks (0.70 ± 0.48 vs. 0.33 ± 0.12; *p* > 0.05). Comparable mineralization was observed in 2D-GPC vs. 2D-BMSC constructs at 4 and 8 weeks (*p* > 0.05). Significantly greater mineralization was observed in 3D-BMSC vs. 3D-GPC constructs at 4 and 8 weeks (*p* < 0.05).

### Cell-Free Constructs Produced Robust Ectopic Mineralization

Substantial mineralization was also observed in the control, i.e., cell-free HPLG-PLATMC, constructs after 4 weeks; μCT analysis revealed comparable MdV/TV to that of 3D-BMSC constructs at 4 weeks (0.97 ± 0.35 vs. 0.92 ± 0.32; *p* > 0.05). Only the cell-free group showed a significant increase in mineralization from 4 to 8 weeks (0.97 ± 0.35 to 1.86 ± 0.60; *p* = 0.003). After 8 weeks, mineralization in the cell-free group was significantly greater than all other groups (*p* < 0.05) (Figures 3A,B).

### Irregular Histological Appearance of Ectopic Mineralization

**FIGURE 4:**
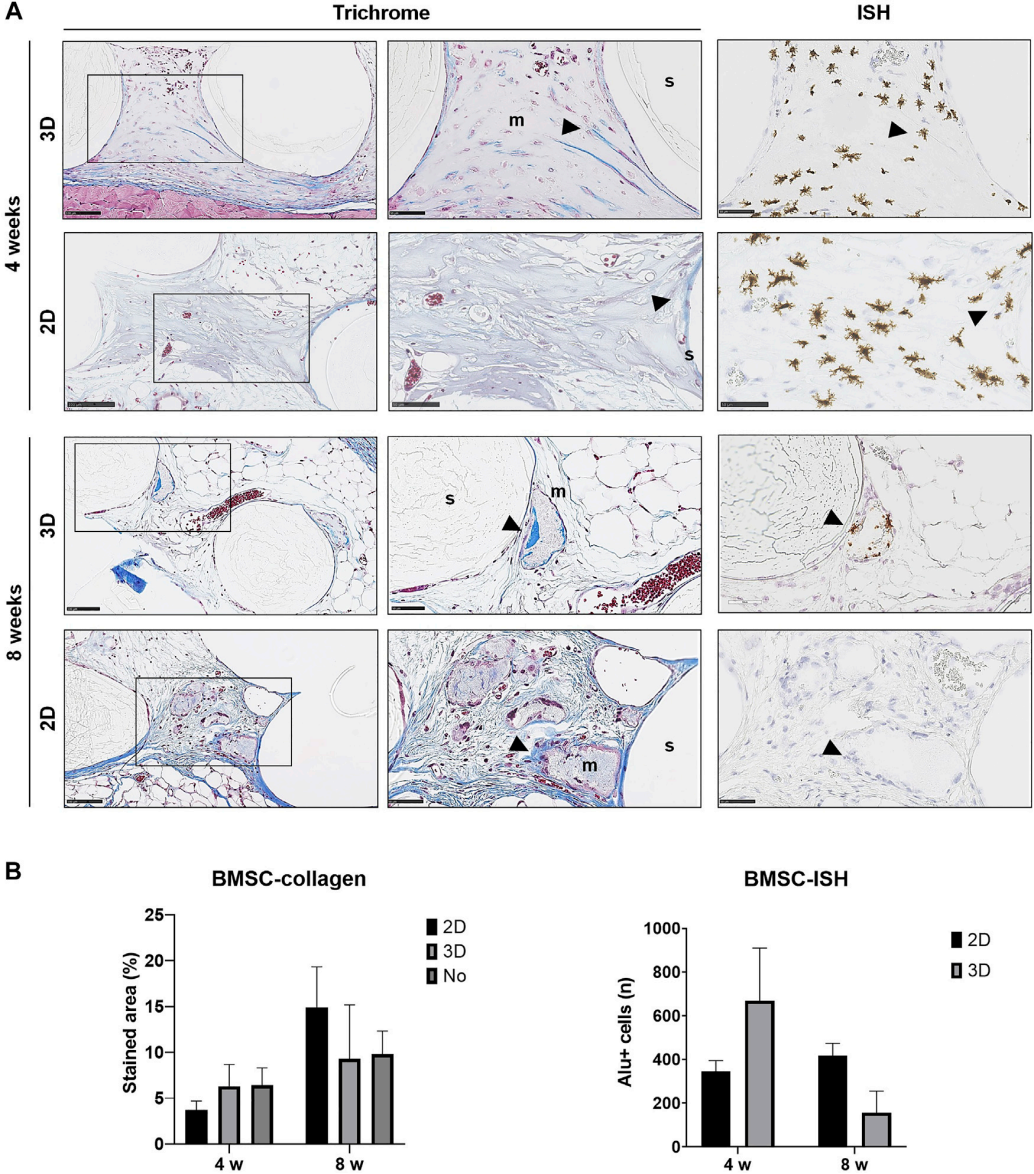
Histology of BMSC constructs. **(A)** Representative images of Trichrome and corresponding ISH stained sections of 2D and 3D BMSC constructs after 4 and 8 weeks; m, mineralization; s, scaffold; wb, woven bone; arrows indicate dense collagen (Trichrome) and Alu + cells (ISH)—except in the ISH section of 2D-GPC at 8 weeks where dense collagen does not correlate with Alu + cells in ISH; scale bars 100 μm. **(B)** Quantification of collagen staining (Trichrome) and Alu + cells (ISH); *n*, total number; data represent means ± SD (*n* = 3).

**FIGURE 5:**
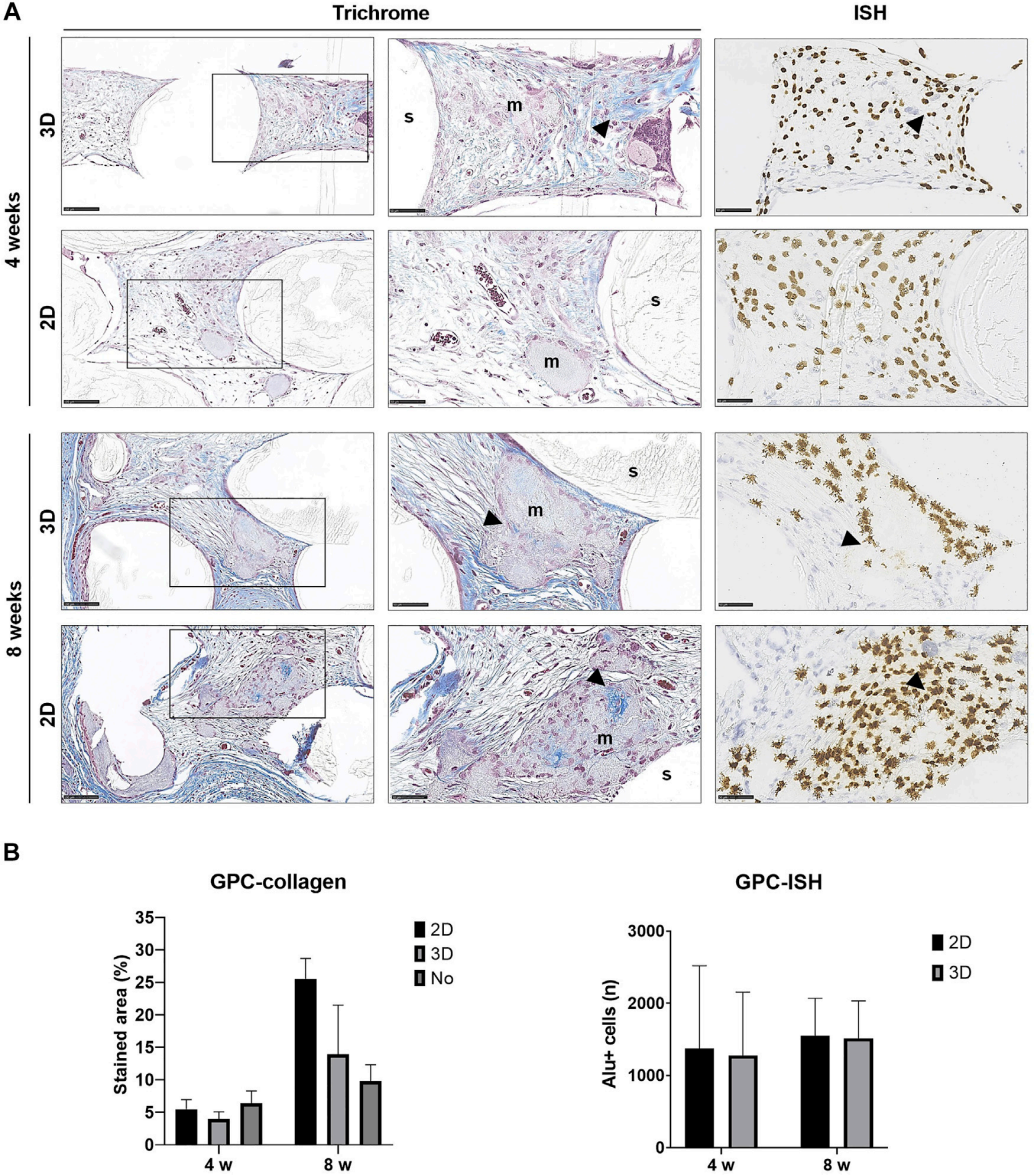
Histology of GPC constructs. **(A)** Representative images of Trichrome and corresponding ISH stained sections of 2D and 3D GPC constructs after 4 and 8 weeks; m, mineralization; s, scaffold; arrows indicate dense collagen (Trichrome) and Alu + cells (ISH); scale bars 100 μm. **(B)** Quantification of collagen staining (Trichrome) and Alu + cells (ISH); n, total number; data represent means ± SD (*n* = 3).

**FIGURE 6:**
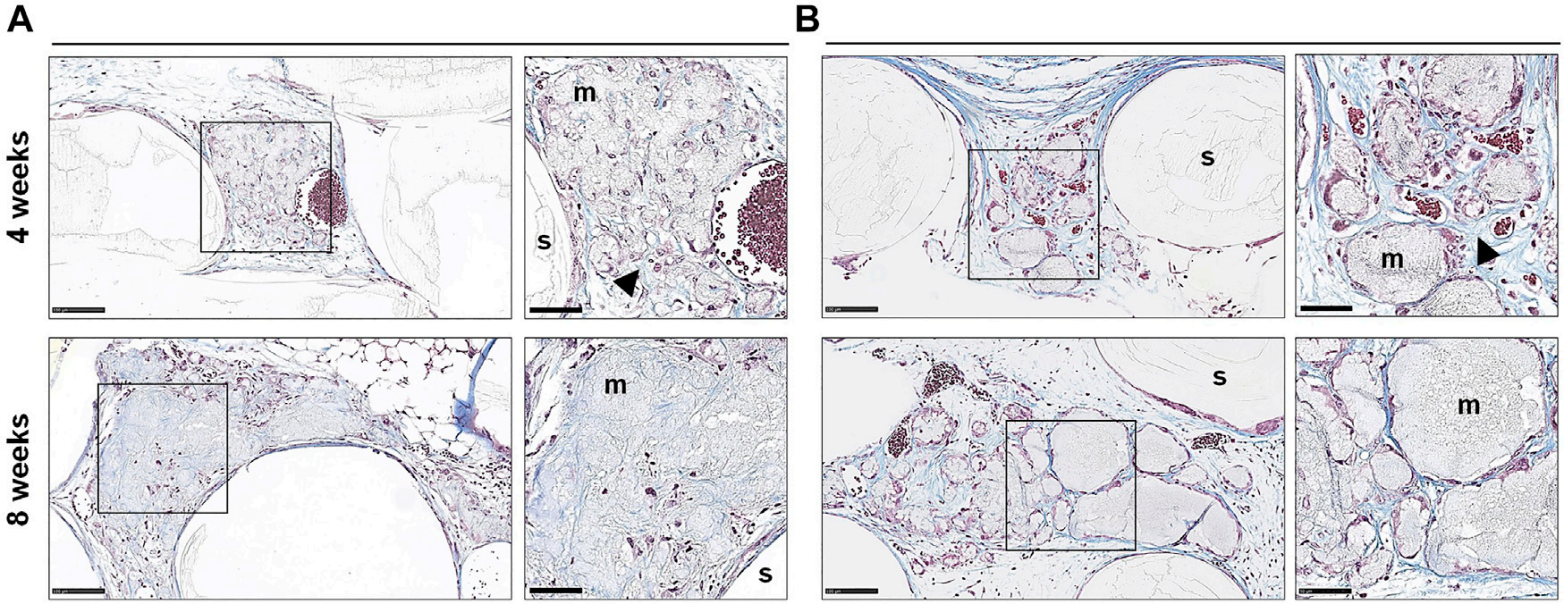
Histology of cell-free constructs. Representative images of Trichrome stained sections of cell-free constructs showing different patterns of mineralization **(A,B)** after 4 and 8 weeks; m, mineralization; s, scaffold; arrows indicate dense collagen; scale bars 100 μm (20x) and 50 μm (40x).

Generally, histological analysis of all explants (cell-loaded and cell-free) revealed fibrous encapsulation of the constructs, with little or no inflammatory cell-infiltrate around the capsules. The scaffold material within the construct was well-defined, could be clearly distinguished from the host tissues and did not indicate any signs of resorption or degradation, even after 8 weeks. The hydrogel between the scaffold pores was degraded and replaced by well-vascularized host tissues (Figures 4–6).

In paraffin-embedded (FFPE) sections, areas of diffuse mineralization were seen along the scaffold margins and between the filaments, often in direct contact with the scaffold. Alizarin red staining of undecalcified FFPE sections confirmed the presence of calcium in the tissues; *Alu +* cells were detected within/surrounding these tissues (Supplementary Figure S1). Presence of collagen was confirmed via Trichrome (blue) staining. After 8 weeks, a trend for higher collagen content was observed in 2D vs. 3D groups of both GPC and BMSC constructs (*p* > 0.05). Overall, no differences in morphology of the mineralized areas or collagen content were observed between cell-loaded and cell-free constructs. A trend for greater collagen and *Alu +* cells was observed in GPC vs. BMSC constructs (*p* > 0.05). In the 3D-BMSC and 3D-GPC groups, the spheroidal form of cell aggregates was retained after 4 weeks and often showed signs of mineralization *en masse* (Supplementary Figure S1).

**FIGURE 7:**
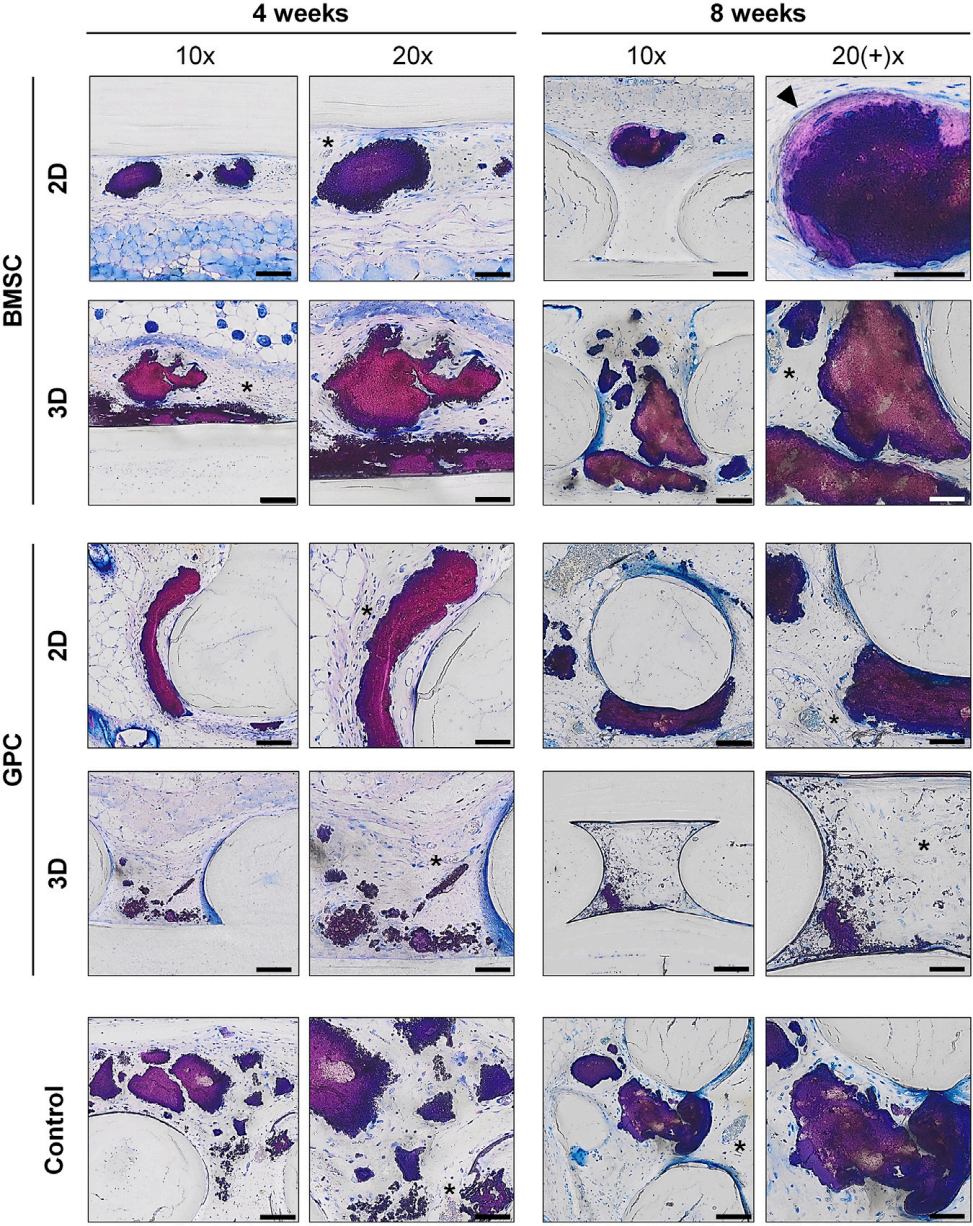
Undecalcified histology. Representative images of BMSC, GPC and control constructs (no cells) after 4 and 8 weeks (thin ground sections, Levi Lazko staining); * indicate blood vessels; arrow indicates the only instance of “bone-like” tissue observed in the study; scale bars 100 μm (10x) and 50 μm (20x).

In FFPE sections, the mineralized areas lacked the organized structure of normal bone tissue, with no evidence of embedded (osteocytes) or lining cells. Similar observations were made in undecalcified RE sections, where the mineralized areas mostly showed an irregular and acellular pattern (Figure 7). Only one instance of organized bone-like tissue with embedded osteocytes was observed in a single specimen from the 2D-BMSC group at 8 weeks. In this case, the new bone was seen to be formed on the surface of an irregular mineralization, which showed roughened borders indicative of resorption (Figure 7).

### Comparable Ultrastructure of Different Mineralization Patterns

**FIGURE 8:**
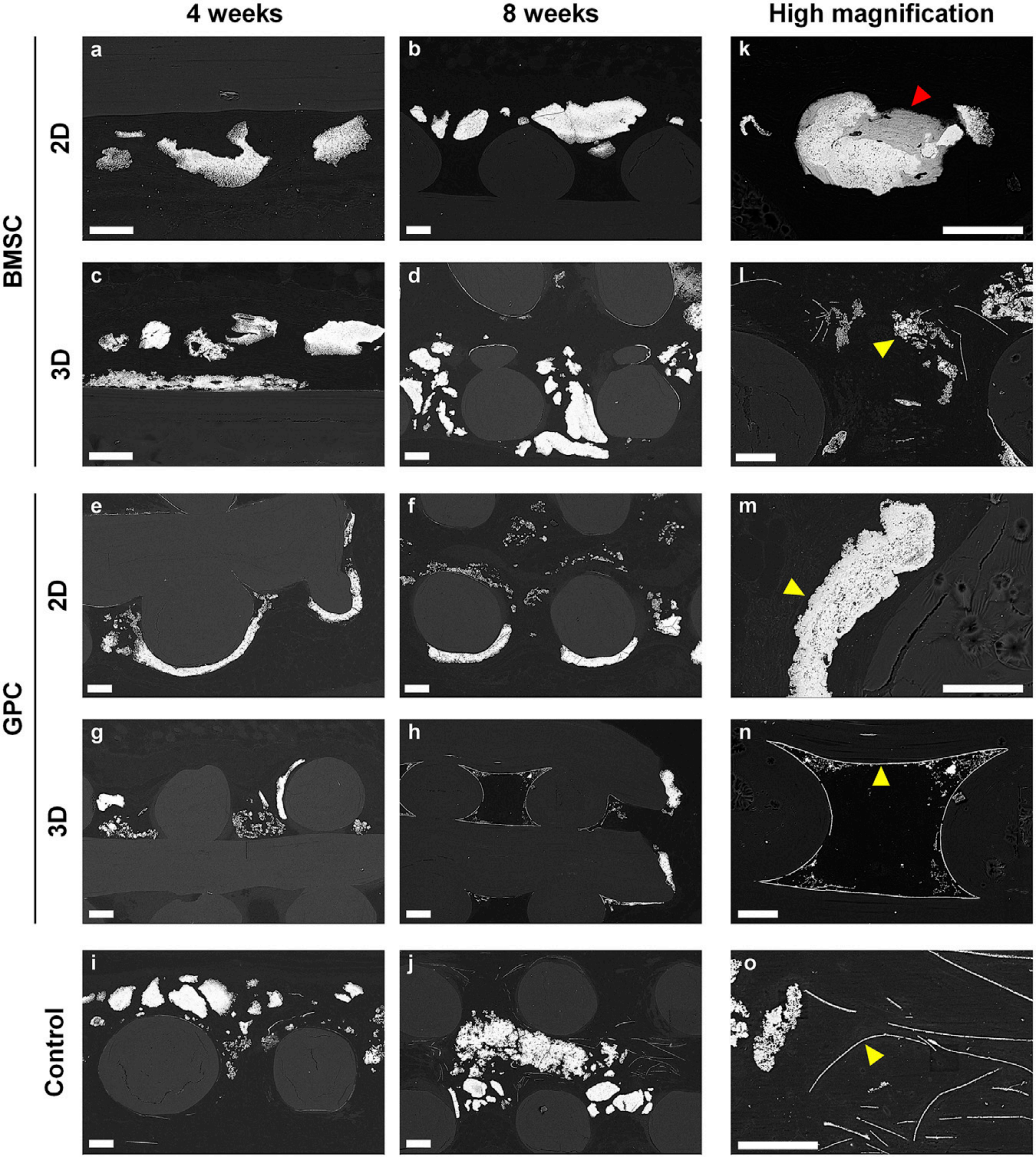
SEM analysis. **(A–J)** Representative images of BMSC, GPC and control constructs (no cells) after 4 and 8 weeks. **(K–O)** Corresponding high magnification images from each group; yellow arrows indicate the different patterns of mineralization: **(K,M)** sheet/plate, **(L)** globular, **(N,O)** filament-like; red arrow indicates the only instance of “bone-like” tissue observed in the study; scale bars 100 μm.

Composition of the different ectopic mineralization patterns in RE sections was further determined via SEM/EDX analysis; SEM and histological images were correlated to analyze specific regions within the mineralized areas. Based on appearance, the different mineralization patterns were categorized as follows (in order of decreasing frequency): globular, plate-like and filament-like (Figure 8). EDX analysis revealed similar compositions in terms of Ca, P and Ca:P ratios between the different mineralization types; average values of Ca, P and Ca:P were 37.31% (range 33.46–41.12%), 17.77% (range 15.73–19.02%) and 2.10 (range 2.02–2.17), respectively. These values were comparable to historical controls of “true” ectopic bone (Supplementary Figure S2).

### Transplanted Cells Detected *in situ* After 8 weeks

Detection of transplanted human cells was performed using ISH for the human specific *Alu* sequence; no *Alu +* cells were detected in cell-free constructs (data not shown). High numbers of *Alu +* cells were detected after 4 and 8 weeks in constructs of both 2D and 3D GPC and BMSC. In 3D GPC/BMSC, cell aggregation was evident even after 8 weeks. *Alu +* cells were uniformly distributed throughout the constructs and associated with markedly denser connective tissue. In several instances, *Alu +* cells were detected within and around the areas of mineralization, although not showing the characteristic lacunae of embedded osteocytes. In BMSC constructs, a trend for greater numbers of *Alu* + cells was observed in the 3D vs. 2D group at 4 but not at 8 weeks (Figure 4B). In GPC constructs, similar numbers of *Alu* + cells were observed in the 3D vs. 2D group at both 4 and 8 weeks (Figure 5B). No significant differences in the numbers of *Alu +* cells were detected between the groups at 4 or 8 weeks (*p* > 0.05).

## Discussion

The present study investigated the ectopic BTE potential of HPLG-PLATMC constructs containing spheroid (3D) or dissociated (2D) BMSC or GPC in a subcutaneous mouse model. The main findings were 1) significantly greater mineralization in constructs of 3D vs. 2D BMSC, 2) comparable mineralization in 2D GPC vs. 2D BMSC, and 3) robust mineralization in cell-free constructs.

In the context of BTE, aggregation of MSC into 3D spheroids has been reported to recapitulate embryonic events during skeletal development and thereby promote their osteogenic differentiation (Hall and Miyake, 2000; Kale et al., 2000). We have recently reported significant upregulations of genes associated with self-renewal and osteogenic differentiation in xeno-free cultures of 3D vs. 2D GPC and BMSC, suggesting a greater potential for *in vivo* osteogenesis (Shanbhag et al., 2020b). Consistently, recent studies have reported superior bone regeneration in rodent orthotopic models when using 3D vs. 2D BMSC encapsulated in Matrigel^®^ (Corning) (Yamaguchi et al., 2014) or alginate-based hydrogels (Ho et al., 2018); similar results were reported for periodontal ligament-derived cells (PDLCs) encapsulated in Matrigel^®^ (Moritani et al., 2018). Conversely, a recent study reported no differences in the healing of mouse femoral defects treated with either 2D or 3D BMSC encapsulated in a commercial fibrin gel (Findeisen et al., 2021). In the present study, significantly greater ectopic mineralization was observed via µCT in 3D vs. 2D BMSC constructs. To our knowledge, only one previous study has reported µCT analysis of ectopic bone formation (Ruminski et al., 2018); another study reported conventional X-ray but not µCT-based assessment of spheroid-hydrogel constructs (Ho et al., 2016). Nevertheless, our findings are supported by previous studies, which reported superior ectopic bone formation by 3D vs. 2D BMSC in calcium phosphate + platelet-rich plasma (PRP) complexes (Chatterjea et al., 2017) or RGD-modified alginate hydrogels (Ho et al., 2016). In the former study (Chatterjea et al., 2017), ectopic bone formation by spheroid BMSC was further enhanced in the presence of PRP, suggesting a synergistic effect of BMSC and platelet-derived growth factors (Shanbhag et al., 2017).

Fibrin- and platelet-based hydrogels, e.g., PRP, have been extensively used as scaffolds for bone regeneration (Soffer et al., 2003). In the present study, a fibrin supplemented HPLG was used to encapsulate the GPC and BMSC spheroids—to preserve their 3D architecture during *in vivo* delivery (Robinson et al., 2016). Indeed, platelet growth factors are known to promote MSC osteogenic differentiation *in vitro* (Kasten et al., 2006; Zhang et al., 2011; Huang et al., 2012; Trouillas et al., 2013; Chatterjea et al., 2017) and bone formation *in vivo* (Kasten et al., 2006; Trouillas et al., 2013; Chatterjea et al., 2017). However, an interesting (and potentially confounding) observation herein was the robust mineralization in cell-free HPLG-PLATMC constructs; after 8 weeks, the greatest µCT-based mineralization was observed in the cell-free group. Since PLATMC is biologically inert, the observed mineralization could be attributed to the HPLG. As already mentioned, although platelet growth factors (PRP) have been shown to enhance MSC-mediated ectopic bone formation, to our knowledge, no studies have detected ectopic bone formation in cell-free fibrin- or PRP-constructs alone (Yamada et al., 2003; Osathanon et al., 2008; Murphy et al., 2015). In context, previous studies have tested “HPL coated” ceramic scaffolds for ectopic and orthotopic bone formation; scaffolds were immersed in HPL for 24 h prior to experiments (Leotot et al., 2013; Bolte et al., 2019). While the HPL coating itself did not promote bone formation, it enhanced the osteogenic potential of BMSC seeded on the scaffolds (Leotot et al., 2013; Bolte et al., 2019). Therefore, whether (and if so, how) human HPL (G) alone can lead to ectopic bone formation requires further investigation.

When comparing cell types herein, comparable ectopic mineralization was observed in constructs of 2D-GPC (MdV/TV 0.70%) vs. 2D-BMSC (0.54%) after 8 weeks. Even constructs of 3D-BMSC (1.03%) did not significantly outperform those of 2D-GPC (0.70%), suggesting that GPC may have the potential to substitute BMSC in future BTE applications. Several studies have investigated *in vivo* bone formation by GPC; some studies have compared the ectopic bone forming potential of GPC and BMSC, of which, three (Fournier et al., 2010; Tomar et al., 2010; Zorin et al., 2014) reported comparable histological “bone formation” between GPC and BMSC (Supplementary Table S1). However, the morphology of mineralized tissues formed by GPC is highly variable in the reported literature—to our knowledge, only few studies have reported regular organized bone tissue with embedded (osteocytes) and/or bone forming cells (osteoblasts) (Supplementary Table S1). These differences in mineralization produced by GPC and BMSC may be explained by the so-called “tissue source variability” (Xu et al., 2017). BMSC are naturally resident in the bone marrow—a specialized tissue niche, and have an inherent propensity for osteogenic differentiation (Hoch and Leach, 2015). Conversely, gingiva is a connective tissue with a mainly supportive function and a large fibroblast-population. Indeed, fibroblasts from various tissues including gingiva are reported to be indistinguishable from BMSC *in vitro* based on the “minimal MSC criteria” (Denu et al., 2016), and the presence of a “true” MSC-like population in gingiva remains to be identified *in vivo* (da Silva Meirelles et al., 2008). Nevertheless, gingiva represents a promising alternative source of progenitor cells for BTE applications.

In contrast to the traditional histological picture of lamellar bone with embedded (osteocytes) and lining cells, an atypical pattern of mineral deposition/precipitation was observed in the constructs herein, regardless of the type or presence of cells. The mineralized areas often appeared as solid masses or aggregates, with no internal lamellar structure or canals containing blood vessels. However, in several instances the mineralized areas revealed the presence of embedded cells, including transplanted BMSC and GPC; in one instance of 2D-BMSC, organized bone-like tissue with embedded osteocytes was observed. A similar pattern of atypical mineralization has previously been reported in rat calvarial defects treated with collagen membranes (Kuchler et al., 2018; Feher et al., 2021). It has been hypothesized that the collagen fibres underwent mineralization via cell-independent mechanisms and thereby served as “scaffolds” for subsequent bone formation (Nudelman et al., 2013) and may explain the observations herein. We observed organized and cellular (osteocyte containing) bone-like tissue around the mineral deposits in one specimen of the 2D-BMSC group at 8 weeks—the mineral deposits showed roughened borders characteristic of surface resorption. This finding supports the hypothesis that the mineral deposits may first undergo resorption and subsequently serve as scaffolds for new bone formation. Other studies have reported dystrophic mineralization of biomaterials in ectopic sites, related to nucleation of calcium-phosphate complexes (Schoen and Levy, 2013; Lotsari et al., 2018). However, extending these hypotheses to the mineralization patterns observed herein is rather speculative, and the exact mechanism(s) of mineralization remains unclear.

*Alu +* GPC and BMSC were detected in the ectopic transplants herein. Detection of transplanted cells via ISH is well established and may assist in understanding the mechanism(s) of *in vivo* bone formation (Mankani et al., 2007; Janicki et al., 2011). It is relevant to note herein that cells (both GPC and BMSC) from pooled donors were used in the present study—to minimize donor-related variation and as a potential future strategy for allogeneic “off-the-shelf” cell therapy. The current literature is inconclusive regarding the mechanism(s) of bone formation by transplanted human MSC—either from independent or pooled donors, i.e., whether this occurs primarily via direct osteogenic differentiation of transplanted cells, paracrine stimulation of host cells, immune modulation, or a combination of factors (Moll et al., 2020). Indeed, *Alu +* cells were identified in the areas of mineralization herein; in several instances, these cells were embedded within the mineralization(s) and/or associated with areas of dense collagen deposition. However, the embedded cells did not demonstrate the well-defined surrounding lacunae characteristic of osteocytes. Previous studies have characterized the role of exogenous cells in ectopic and orthotopic bone formation. For example, transplantation of allogeneic BMSC in immunocompetent mice revealed immune modulation rather than osteoblastic differentiation in one study (Tsujigiwa et al., 2013; Takabatake et al., 2018). In another study, no transplanted human BMSC could be detected in ectopic mouse transplants beyond 2 weeks, despite robust bone formation at 8 weeks (Gamblin et al., 2014). These reports further suggest that transplanted BMSC contribute to bone formation via stimulation of tissue-resident progenitor cells rather than direct differentiation into osteoblasts (Tsujigiwa et al., 2013; Takabatake et al., 2018). Indeed, the type and immune status of the animal-model may also influence *in vivo* osteogenesis (Garske et al., 2020). Based on previous literature, we selected the athymic “nude” mouse model (Scott et al., 2012), where the absence of functional T lymphocytes (and partial defect of B cells) allows for xenogeneic transplantation of human cells without immune rejection. Others have reported favourable ectopic bone formation by human BMSC in NMRI-nude (Brennan et al., 2014; Gamblin et al., 2014) and NOD-SCID mice (Suliman et al., 2019), which present certain differences in immune status compared to our mouse model. Nevertheless, the exact mechanism(s) of osteogenesis and/or mineralization by xeno-transplanted BMSC in immunocompromised rodent models remains to be elucidated.

## Conclusion

In summary, ectopic implantation of the various HPLG-PLATMC constructs revealed significantly greater mineralization in those with 3D-BMSC vs. 2D-BMSC and comparable mineralization in those with 2D-GPC vs. 2D-BMSC. However, the effect of cell transplantation was confounded by that of HPLG, based on the robust mineralization observed in cell-free constructs. Although transplanted GPC and BMSC were detected *in situ* after 8 weeks, their direct contribution to mineralization could neither be confirmed nor excluded. GPC represents a promising alternative to BMSC for BTE. The HPLG-PLATMC constructs herein represent promising and clinically relevant scaffolds for BTE applications.

## Data Availability

The raw data supporting the conclusion of this article will be made available by the authors, without undue reservation.
