# Corrigendum: Ectopic Bone Tissue Engineering in Mice Using Human Gingiva or Bone Marrow-Derived Stromal/Progenitor Cells in Scaffold-Hydrogel Constructs

**DOI:** 10.3389/fbioe.2021.831669

**Published:** 2022-01-04

**Authors:** Siddharth Shanbhag, Carina Kampleitner, Samih Mohamed-Ahmed, Mohammed Ahmad Yassin, Harsh Dongre, Daniela Elena Costea, Stefan Tangl, Mohamad Nageeb Hassan, Andreas Stavropoulos, Anne Isine Bolstad, Salwa Suliman, Kamal Mustafa

**Affiliations:** ^1^ Center for Translational Oral Research (TOR), Department of Clinical Dentistry, Faculty of Medicine, University of Bergen, Bergen, Norway; ^2^ Department of Immunology and Transfusion Medicine, Haukeland University Hospital, Bergen, Norway; ^3^ Ludwig Boltzmann Institute for Traumatology, The Research Center in Cooperation with AUVA, Vienna, Austria; ^4^ Karl Donath Laboratory for Hard Tissue and Biomaterial Research, University Clinic of Dentistry, Medical University of Vienna, Vienna, Austria; ^5^ Austrian Cluster for Tissue Regeneration, Vienna, Austria; ^6^ Gade Laboratory for Pathology, Department of Clinical Medicine, Faculty of Medicine, University of Bergen, Bergen, Norway; ^7^ Centre for Cancer Biomarkers (CCBIO), Faculty of Medicine, University of Bergen, Bergen, Norway; ^8^ Department of Periodontology, Faculty of Odontology, Malmö University, Malmö, Sweden; ^9^ Division of Regenerative Medicine and Periodontology, University Clinics of Dental Medicine, University of Geneva, Geneva, Switzerland

**Keywords:** xeno-free, platelet lysate, MSc, spheroid culture, bone tissue engineering

“Mohamad Nageeb Hassan” was not included as an author in the published article. The corrected **Author Contributions** Statement appears below.

“SSh and KM conceived and designed the study. SSh performed the experiments, data collection, data analysis and drafted the manuscript. CK, SM-A, MY, HD, DE, ST, and MH assisted with data collection, data analysis/interpretation and drafting the manuscript. AS, AB, SSu, and KM assisted with data analysis/interpretation and drafting the manuscript. All authors read and approved the final version of the manuscript.”

The authors apologize for this error and state that this does not change the scientific conclusions of the article in any way. The original article has been updated.

