# Supplementary material for: Ectopic Bone Tissue Engineering in Mice Using Human Gingiva or Bone Marrow-Derived Stromal/Progenitor Cells in Scaffold-Hydrogel Constructs
Source: Front Bioeng Biotechnol. 2021 Nov 30;9:783468. doi: 10.3389/fbioe.2021.783468 (PMC8670384; doi:10.3389/fbioe.2021.783468)
Supplement: Supplementary file 1 [file DataSheet1.docx]

**Supplementary data**

1. ***Supplementary figures***
   1. **Supplementary figure 1: Alizarin red staining of histological sections**


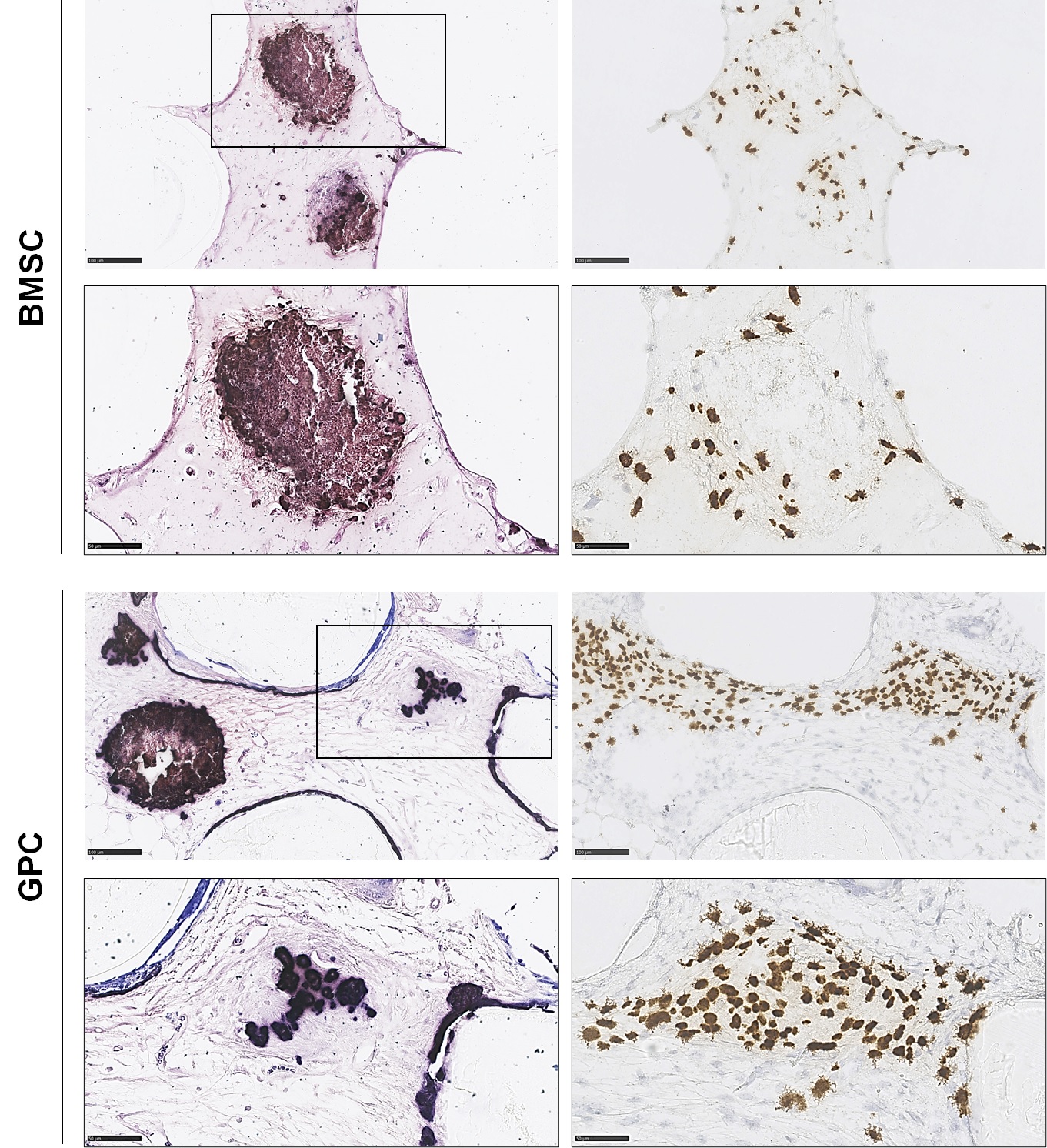


Low (above, scale bar 100 µm) and high (below, scale bar 50 µm) magnification images of Alizarin red staining (left) and corresponding ISH staining of undecalcified FFPE sections of 3D-BMSC and 3D-GPC constructs after 4 weeks; red staining indicates calcium deposition. Note that the spheroidal form of aggregates is preserved, and spheres demonstrate *in situ* mineralization. ISH shows transplanted cells in the mineralized tissues.

- 1. **Supplementary figure 2: Positive control samples for SEM/EDX analysis**


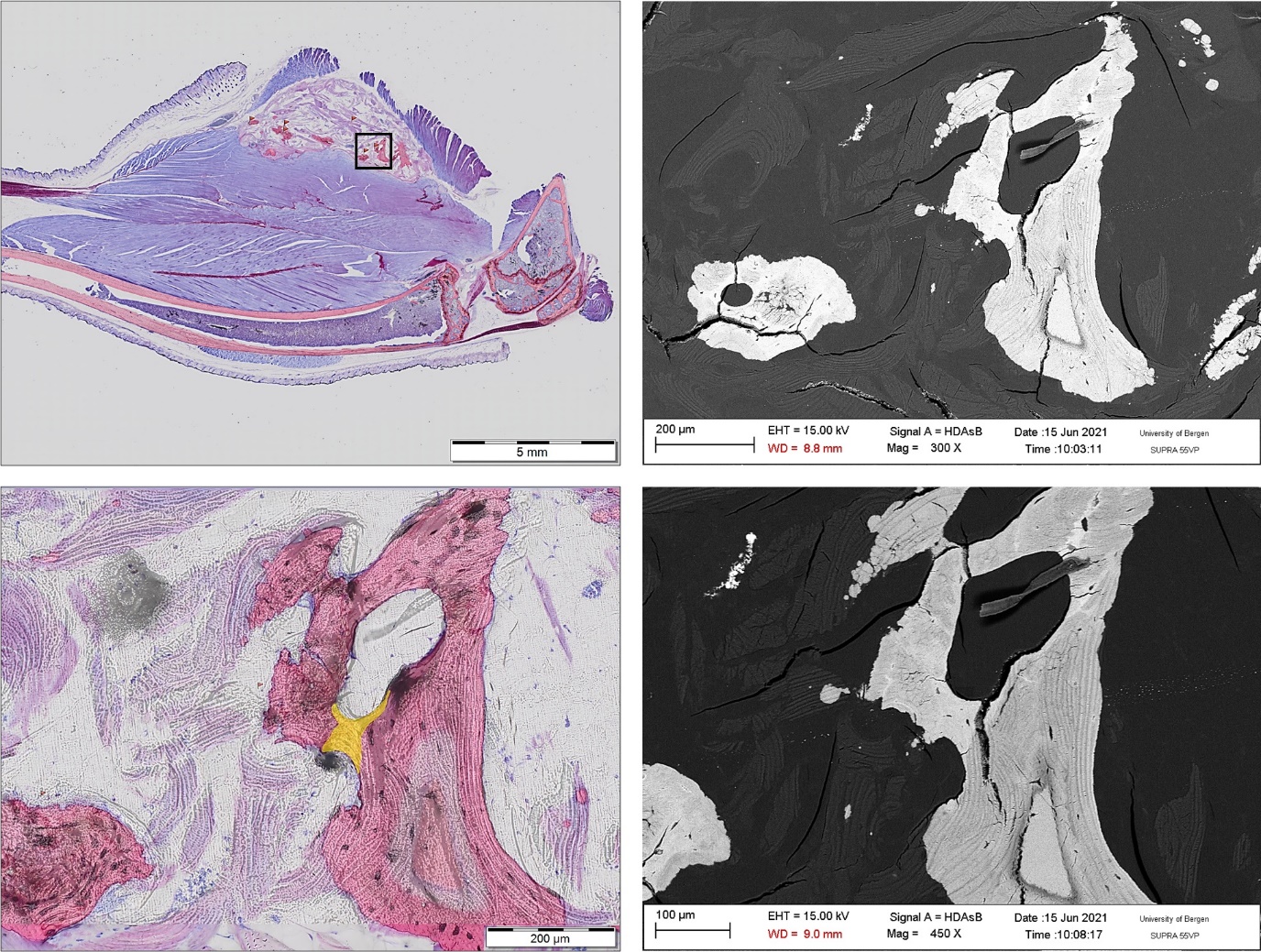


Histological (left) and SEM (right) images of ectopic bone formation in mouse intramuscular sites from a previous study (unpublished) used as a positive control in the present study; note the presence of typical bone with lamellar structure and embedded osteocytes. The EDX values of mineral tissues in the cell(-free) constructs were comparable to those of the positive control.

1. ***Supplementary tables***
   1. **Supplementary table 1: Summary of studies reporting ectopic transplantation of GPC**

| **Study** | **GPC** | **Other cells** | **Methods** | **Outcome (“bone”)** | **Histo. appear-ance*** |
| --- | --- | --- | --- | --- | --- |
| Fournier et al. 2010 (1) | GMPC, 2X10^6^ | GF, BMSC | HA  8 w, Nude mice | Histo: GMPC = BMSC | Irregular |
| Tomar et al. 2010 (2) | GMSC, 10^6^, osteo 10d | BMSC | BCP  10 w, Scid mice | Histo: GMSC = BMSC | Irregular |
| Wang et al. 2011 (3) | GMSC, 5x10^6^ | - | Collagen gel  6 w, Nude mice | Histo: no bone, GMSC or gel  IHC-Opn: GMSC > gel | Irregular |
| Zorin et al. 2014 (4) | GMSC, 5x10^6^ | BMSC | OCP, TCP  3,4,7 w, Nude mice | Histo/IHC: GMSC = BMSC | Irregular |
| Yang et al. 2013 (5) | GMSC, sheets, osteo 7d+ (+/- TNFa, IL1b) | PDLSC | BS  8 w, Scid mice | Histo: PDLSC > GMSC; No inf. > inf. | Regular |
| Ge et al. 2012 (6) | GMSC, healthy or inf., 5x10^6^ | PDLSC | BCP + fibrin gel  8 w, Nod-Scid mice | Histo: PDLSC > GMSC (no bone) | Irregular |
| Shi et al. 2019 (7) | GMSC +/- TGFb-inh., 2x10^6^ | - | HA  12 w, BALB/c mice | Histo: GMSC+TGFb-inh > GMSC > HA-only | Regular |

* Histo. appearance, morphology of “bone” or mineralized tissues as shown in the published histological images as compared to the typical (regular) histological appearance of bone, i.e., lamellar structure with embedded osteocytes.

GMPC, gingiva-derived progenitor cells; GMSC, gingiva-derived MSCs; GF, gingival fibroblasts; BMSC, bone marrow mesenchymal stromal cells; PDLSC, PDL-derived MSCs (terminology in original studies); osteo, osteogenic induction; histo: histology; no bone, no bone/mineralized tissue formation; IHC-OPN, immunohistochemistry for osteopontin; inf., inflamed; TGFβ-inh., TGFβ inhibitor; HA, hydroxyapatite; OCP, octa-calcium phosphate; BCP, biphasic calcium phosphate; TCP, tri-calcium phosphate; BS, bone substitute; d, days; w, weeks.

**References**

1. Fournier BP, Ferre FC, Couty L, Lataillade JJ, Gourven M, Naveau A. Multipotent progenitor cells in gingival connective tissue. Tissue Eng Part A. 2010;16:2891-9.

2. Tomar GB, Srivastava RK, Gupta N, Barhanpurkar AP, Pote ST, Jhaveri HM. Human gingiva-derived mesenchymal stem cells are superior to bone marrow-derived mesenchymal stem cells for cell therapy in regenerative medicine. Biochem Biophys Res Commun. 2010;393:377-83.

3. Wang F, Yu M, Yan X, Wen Y, Zeng Q, Yue W. Gingivaderived mesenchymal stem cell-mediated therapeutic approach for bone tissue regeneration. Stem Cells Dev. 2011;20:2093-102.

4. Zorin VL, Komlev VS, Zorina AI, Khromova NV, Solovieva EV, Fedotov AY, et al. Octacalcium phosphate ceramics combined with gingiva-derived stromal cells for engineered functional bone grafts. Biomed Mater. 2014;9(5):055005.

5. Yang H, Gao LN, An Y, Hu CH, Jin F, Zhou J. Comparison of mesenchymal stem cells derived from gingival tissue and periodontal ligament in different incubation conditions. Biomaterials. 2013;34:7033-47.

6. Ge S, Mrozik KM, Menicanin D, Gronthos S, Bartold PM. Isolation and characterization of mesenchymal stem cell-like cells from healthy and inflamed gingival tissue: potential use for clinical therapy. Regen Med. 2012;7:819-32.

7. Shi A, Heinayati A, Bao D, Liu H, Ding X, Tong X, et al. Small molecule inhibitor of TGF-beta signaling enables robust osteogenesis of autologous GMSCs to successfully repair minipig severe maxillofacial bone defects. Stem Cell Res Ther. 2019;10(1):172.
